# Research Progress on Modification Strategies of Nanoscale Zero-Valent Iron and Its Application in the Removal of Organic Pollutants

**DOI:** 10.3390/nano16171090

**Published:** 2026-08-31

**Authors:** Jing Wei, Liying Ren, Xilei Wang, Guoshuai Gao, Xin Lin

**Affiliations:** 1 College of Resources and Environment, Linyi University, Linyi 276000, China; 2 Ecological Environment Technical Service Center of Zhangdian District, Zibo 255000, China

**Keywords:** nanoscale zero-valent iron, surface modification, organic pollutants, degradation mechanism

## Abstract

Nanoscale zero-valent iron (nZVI) exhibits great potential in the field of organic pollutant remediation due to its strong reducibility, high specific surface area and unique core–shell structure. However, pristine nZVI has inherent drawbacks including severe particle aggregation, surface passivation and poor electron selectivity, which greatly restrict its practical remediation performance. To improve the reactivity of nZVI, researchers have developed multiple modification approaches that significantly improve the dispersibility, stability and reactivity of nZVI. This review summarizes the main nZVI modification strategies, including metal modification, surface coating, carrier loading, sulfidation modification and biological integration. The advantages and limitations of each modification method are compared. Furthermore, the underlying removal mechanisms of modified nZVI toward typical organic pollutants are elaborated, covering direct reduction, advanced oxidation and synergistic degradation pathways. Key factors governing the degradation efficiency of modified nZVI are subsequently analyzed. Finally, existing bottlenecks for practical implementation and future research perspectives are proposed.

## 1. Introduction

With the acceleration of industrialization, organic pollutants such as halogenated organic compounds, nitroaromatic hydrocarbons, dyes, pesticides and antibiotics continuously accumulate in soil and groundwater environments, which poses a severe threat to human health and ecological security [1,2]. Traditional physical remediation methods are costly, chemical oxidation methods tend to generate toxic intermediates, and bioremediation is limited by long treatment periods and unsatisfactory removal efficiency. The development of nanotechnology has provided new material tools for organic pollution remediation [3]. Among the various functional materials for pollution remediation, nanoscale zero-valent iron (nZVI) has emerged as a research hotspot in environmental remediation due to its unique advantages: exceptional electron donation capability, high specific surface area, low raw material cost and ability to simultaneously treat organic–heavy metal composite pollutants [4].

Nanoscale zero-valent iron refers to elemental iron particles with a particle size ranging from 1 to 100 nm. It features a typical core–shell structure consisting of a metallic Fe^0^ core encapsulated by an iron (hydr)oxide shell. This unique structure endows nZVI with multiple functions. The Fe^0^ core can act as a strong reducing agent (E^0^ = −0.44 V), which can reductively detoxify organic pollutants through electron transfer-mediated reductive reactions [5]. Meanwhile, nZVI can activate molecular oxygen or persulfate to generate highly reactive radicals, which enables the mineralization and degradation of organic compounds via advanced oxidation pathways. The iron (hydr)oxide shell on its surface gives it adsorption properties. As a result, nZVI has attracted extensive attention in the field of organic pollution remediation [6,7,8]. This review mainly focuses on authentic nZVI with a particle size between 1 and 100 nm. A small set of representative submicron zero-valent iron materials are also discussed when they provide valuable insights for nanoscale modification.

Since nZVI was first applied for groundwater halogenated hydrocarbon remediation in the 1990s, researchers have conducted extensive studies on its performance optimization and application mechanisms [9]. However, nZVI still faces challenges in practical applications, including particle aggregation, surface passivation, poor electron selectivity and limited mobility in subsurface environments. All of these severely constrain its actual remediation performance [10]. To overcome these drawbacks, researchers have developed multiple nZVI modification strategies, including bimetallic doping, surface coating, carrier loading, sulfidation modification and biological integration. Different modification methods can substantially alter the surface adsorption properties of nZVI, including specific surface area, pore structure, surface charge and pollutant affinity. These properties are critical prerequisites for the subsequent redox degradation reactions. These strategies have significantly improved the removal efficiency of organic pollutants [11,12,13,14].

Several previous reviews have covered nZVI synthesis, modification, characterization and pollutant removal behaviors [7,8,10,15]. But each review focuses on specific modification strategies and pollutants. This review focuses on modification strategies of nZVI and their application mechanisms in organic pollutant removal, with an emphasis on the research advancements conducted between 2020 and 2026. Several foundational pre-2020 references are supplemented to complete the background knowledge. This review summarizes the advantages and drawbacks of different modification methods and provides an analysis of the degradation mechanisms of typical organic pollutants by modified nZVI, aiming to provide references for future research and practical applications in this field.

## 2. Modification Strategies of Nano Zero-Valent Iron

**Figure 1:**
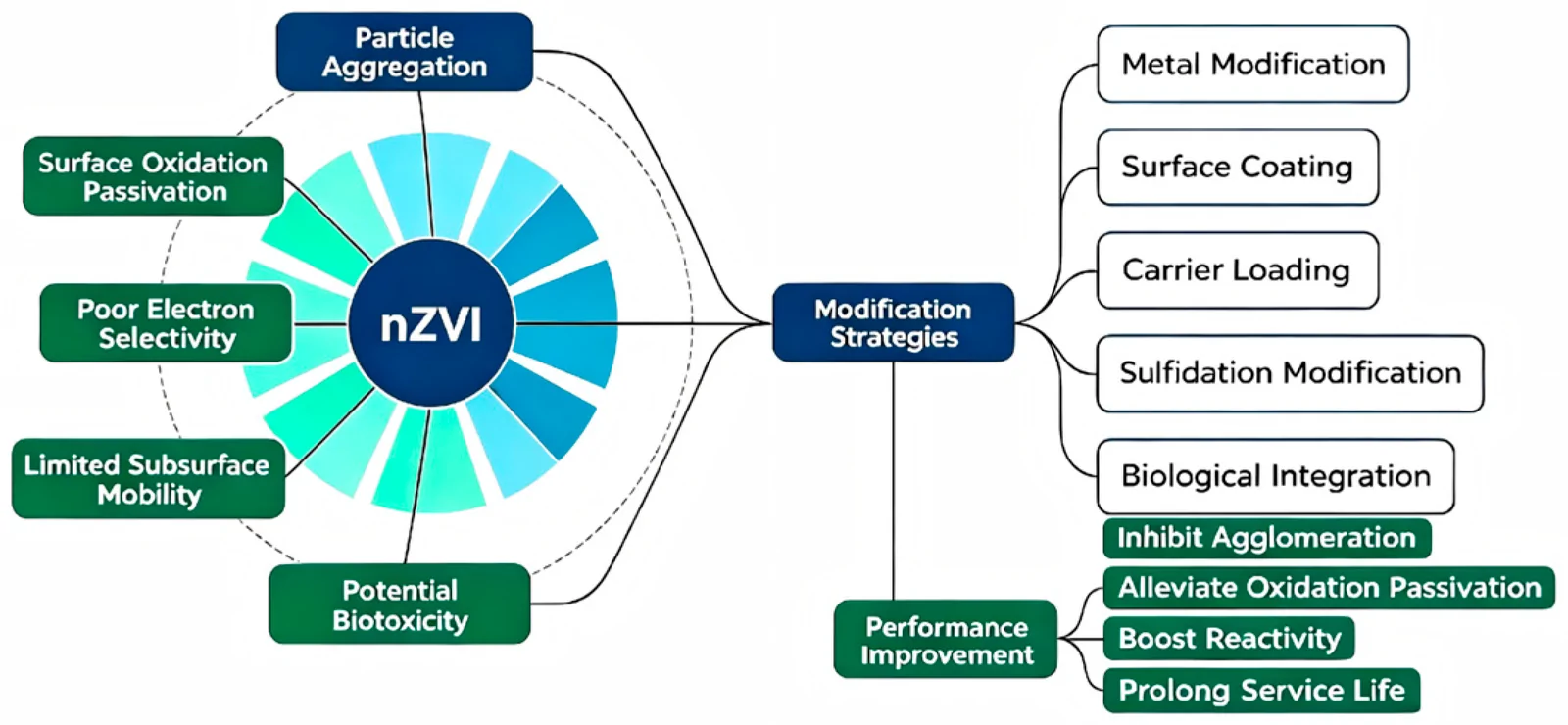
Drawbacks and modification strategies of nZVI.

Pristine nZVI has inherent drawbacks, such as aggregation caused by magnetism, susceptibility to surface oxidation and passivation and poor electron selectivity. These limitations reduce its removal efficiency for organic pollutants. In recent years, researchers have developed a variety of modification strategies to address these limitations. The core principle of nZVI modification lies in regulating its surface structure and electronic properties through physical, chemical or biological methods to inhibit aggregation and oxidative passivation, thereby enhance reactivity and reaction longevity. Based on the type of modification method, these strategies are mainly classified into metal modification, surface coating, carrier loading, sulfidation modification and biological integration (Figure 1). Each type of modification strategy possesses distinct characteristics. The following sections provide a detailed elaboration based on relevant research findings.

### 2.1. Metal Modification

**Figure 2:**
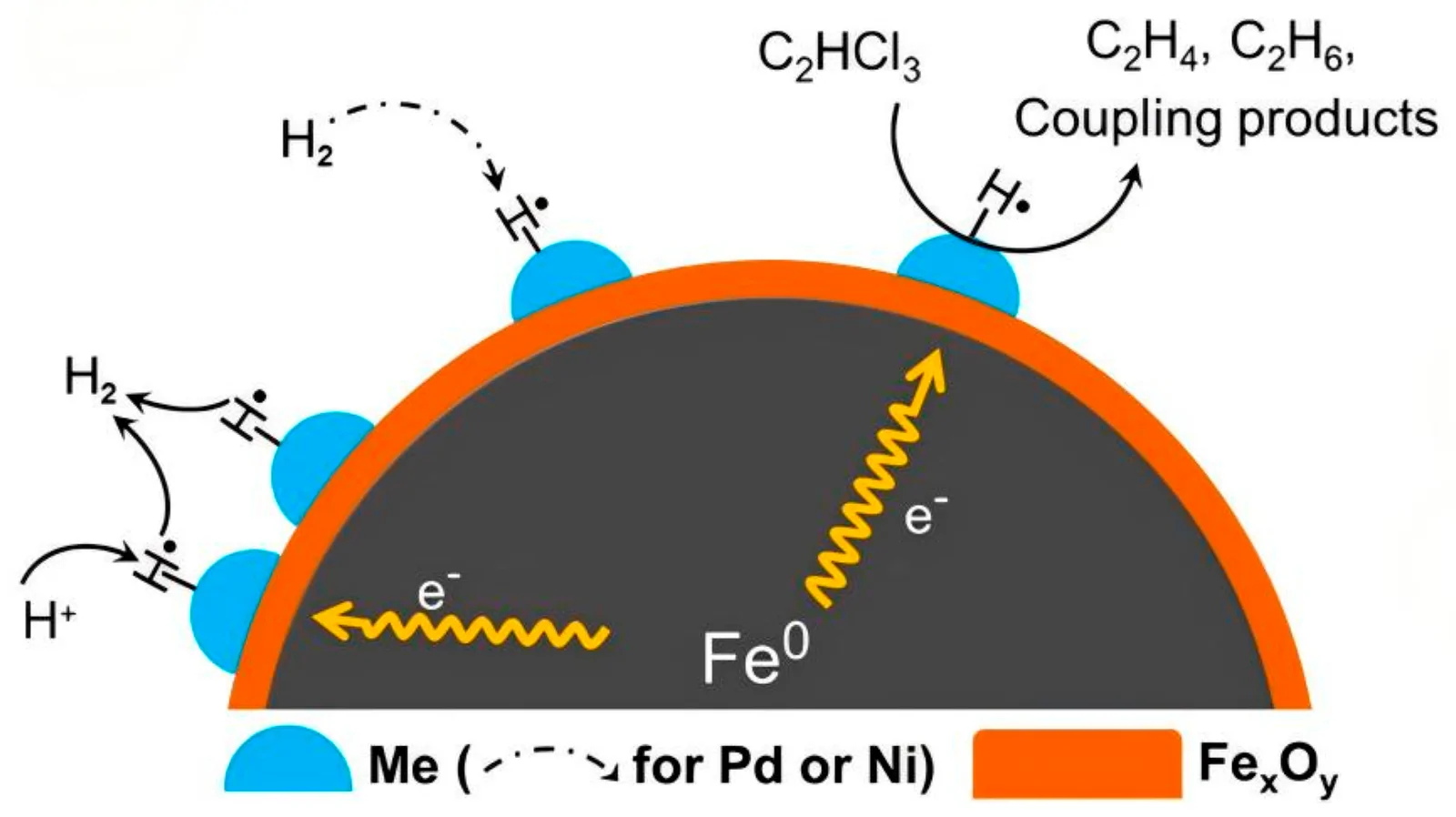
Schematics of the proposed TCE dechlorination and HER on Fe-Me. Reproduced with permission from reference [16], Copyright 2018 American Chemical Society.

Metal modification is among the most widely employed strategies for nZVI modification. The underlying principle is to form composites between nZVI and noble/transition metals. This leverages the synergistic effect between metals to regulate electron transfer efficiency, inhibit oxidative passivation and enhance the catalytic degradation capacity for organic pollutants (Figure 2) [16]. The theoretical basis behind metal modification originates from the corrosion theory of galvanic cells. Through the deposition of metals such as Pd, Cu, Ni, Ag or Pt onto nZVI particles surface, a Fe^0^-based heterometallic microgalvanic cell system is established. Since these secondary metals exhibit higher standard electrode potential than Fe, Fe^0^ serves as the anode and continuously releases electrons, while heterometallic sites act as cathodes to accumulate electrons and facilitate their directional transfer toward organic pollutants. Such a process significantly reduces electron transfer resistance, suppresses unfavorable side reactions between electrons and water molecules or dissolved oxygen and improves the selectivity of electron utilization [8]. Meanwhile, abundant defect sites generated at bimetallic interfaces can activate dissolved oxygen or persulfate to produce oxidative free radicals, realizing synergistic redox degradation of organic contaminants. This strategy can be further divided into noble metal modification and transition metal modification according to the type of metal.

Noble metal modification primarily functions via catalytic effects. It reduces the electron transfer resistance on the nZVI surface, accelerates the reductive degradation of organic pollutants and inhibits the oxidative passivation of nZVI. Pd is the most commonly used noble metal modifier due to its high catalytic activity and favorable stability. In Pd/nZVI composites, Pd serves as an electron transfer station to accelerate electron transfer from Fe^0^ on the nZVI surface. This prevents the oxide layer from thickening caused by electron accumulation on the surface and catalyzes the reduction reaction of trichloroethene (TCE) [17]. nZVI/Pd achieved complete degradation of pollutants within 24 h, whereas pristine nZVI only reached only 6%, 57% and 26% degradation for 1,2-dichloroethene (DCE), TCE and perchloroethene (PCE), respectively [18]. However, the high cost of noble metals and corresponding high preparation expenses limit their large-scale engineering applications.

Transition metal modification aims to reduce modification costs and improve the reactivity of nZVI. Its reaction mechanisms mainly include synergistic reduction, catalytic oxidation and aggregation inhibition. Cu is the most widely used transition metal modifier due to its low cost and favorable catalytic activity [19]. In the Cu/nZVI galvanic cell system, Fe is oxidized at the anode, whereas Cu serves as the cathode to accelerate electron transfer. Additionally, Cu catalyzes the decomposition of H_2_O_2_ generated by nZVI into •OH. This enables the catalytic oxidative degradation of organic pollutants [20]. As another transition metal catalyst, Ni, forms bimetallic system with nZVI and generates a synergistic effect. The Fe-Ni galvanic cell accelerates Fe^0^ electron release, and Ni provides active sites for reductive catalysis. Ni-modified nZVI exhibits enhanced reactivity toward nitrobenzene. For chlorinated organic compound degradation, nZVI/Ni and nZVI/Pd show comparably excellent catalytic performance, which suggests that Ni can serve as a cost-effective alternative for Pd. However, Pd still retains comprehensive advantages in terms of catalytic activity and stability [18].

Metal modification presents a number of distinct core advantages. It can construct galvanic cells and accelerate the directional transfer of electrons, which increases the degradation rate by several-fold. It can simultaneously activate oxidants at cathode metal sites and enable reduction–oxidation synergistic degradation. Their simple preparation process only requires a single substitution reaction for modification, which makes it easy to implement in laboratories.

Its limitations should also be noted. Modification with noble metals is costly and unsuitable for large-scale on-site remediation. Common metal-modified materials such as Cu and Ni are prone to leach metal ions in acidic water conditions, which leads to secondary heavy metal pollution. Bimetallic particles still suffer from agglomeration, and rapid accumulation of surface passivation layers occurs during long-term operation, which reduces their reaction efficiency.

### 2.2. Surface Coating Modification

Surface coating refers to applying a polymer or surfactant layer on the nZVI surface to prevent particle aggregation. Commonly used polymers include polyvinylpyrrolidone (PVP), carboxymethyl cellulose (CMC), chitosan (CS) and polyacrylic acid (PAA). These polymers have excellent water solubility and biocompatibility and can bind to the nZVI surface through functional groups to form a stable coating layer [21,22]. The coating material optimizes nZVI performance from both physical and chemical perspectives. Physically, the coating layer isolates van der Waals forces among nanoparticles, inhibits particle aggregation and blocks dissolved oxygen in water, thus delaying the oxidation and passivation of the Fe^0^ core. Chemically, the coating introduces functional groups including hydroxyl groups, carboxyl groups and hydrophobic cavities, which provide specific adsorption sites for nZVI. These sites enable nZVI to selectively capture hydrophobic organic pollutants in water and shorten the reaction distance between the pollutants and iron active interface [10,23].

PVP is one of the most commonly used polymer modifiers. The pyrrolidone groups in its molecules can form coordination bonds with Fe atoms on the nZVI surface, which create a protective film on the nZVI surface. Meanwhile, the steric hindrance effect between PVP molecules effectively prevents particle aggregation. To inhibit atmospheric oxidation of nZVI and improve its reusability and electron mobility, researchers have prepared CMC-PVP-nZVI/Pd nanocomposites. This modified material achieved a 99% cyanide removal efficiency at an initial cyanide concentration of 100 mg L^−1^, which is 1.8 times higher than that of unmodified nZVI [24].

Chitosan, as a natural biopolymer, is environmentally friendly and biodegradable. The amino and hydroxyl groups in its molecules can bind to the nZVI surface, while the hydrophilicity of CS enhances nZVI dispersibility. Additionally, CS itself possesses a certain adsorption capacity for organic pollutants, which enable an adsorption–degradation synergy. Hydroxyl groups on the CS surface effectively prevent nanoparticle aggregation and significantly improve its dispersibility and reactivity. CS@nZVI composite achieved an 85.53% COD removal efficiency and 89.73% decolorization efficiency for textile wastewater and retained good regenerability after five reuse cycles [25]. By loading nZVI onto CS-modified ZSM-5 zeolite, the dispersibility and stability of nZVI are effectively improved. nZVI@C-ZSM composite achieved 97.75% tetracycline (TC) removal efficiency within 60 min, which is twice that of unmodified nZVI [26].

PAA anchors onto nZVI’s surface through its carboxyl groups to form stable complexes and improve the dispersibility and stability of nZVI. XRD and TEM characterization confirmed that the PAA coating protects the Fe core. In the presence of coexisting anions and humic acid, PAA-nZVI exhibited 3-fold higher TCE reduction capacity than inorganically modified nZVI [27].

Cyclodextrin (CD) polymer coatings have emerged as a breakthrough direction in coating modification in recent years. The hydrophobic cavities of CD play a crucial role in pollutant removal. Based on cavity size among α-, β- and γ-cyclodextrins, these molecules can selectively form complexes with persistent organic pollutants such as cycloaromatics, phenols and antibiotics that have matching molecular weights [28]. By incorporating CD-crosslinked polymers to nZVI (CDP-nZVI), the crosslinked polymer network confines the nZVI particles within three-dimensional pores, which completely prevents aggregation and increases local interfacial pollutant concentrations by several orders of magnitude. Experiments demonstrated that CDP-nZVI can effectively treat naphthalene-contaminated river water, achieving a degradation efficiency exceeding 90% after five regeneration cycles [23].

Surface coating utilizes steric hindrance and electrostatic repulsion to effectively reduce magnetic attraction, suppress particles aggregation and enhance their stability and dispersibility in aqueous solutions. Additionally, the oxygen-containing functional groups introduced by the modifier act as pollutants pre-enrichment sites and form strong complexes, which improves the pollutant removal efficiency. However, the dosage of the coating agent is a critical factor that determines the modification efficacy. Insufficient coating cannot form complete protective layers and fails to realize anti-aggregation or anti-passivation. Excessive dosage generates dense non-porous films that block Fe^0^ electron export and deteriorate degradation activity. Therefore, a balance between dispersibility and reactivity is indispensable. When selecting coating materials for practical applications, existing water chemistry conditions must be taken into consideration.

### 2.3. Carrier Loading Modification

Carrier loading modification involves the in situ growth and impregnation of nZVI nanoparticles into the pores of porous carriers. It achieves performance enhancement via the three-dimensional porous framework of the carrier. The pores of the carrier can physically isolate nZVI particles, fundamentally prevent nanoparticle aggregation and fully expose Fe^0^ active sites. The porous carrier with large specific surface area and abundant functional groups pre-adsorbs organic pollutants via pore filling, electrostatic adsorption and hydrogen bonding interactions. This creates a localized high-pollutant concentration zone at the nZVI interface and significantly accelerates reaction kinetics. Furthermore, carrier frameworks buffer pH fluctuations, isolate partial dissolved oxygen, delay nZVI surface passivation and endow materials with recoverability to facilitate magnetic separation and solid–liquid separation [28,29].

At present, carriers can be divided into three main categories: carbon-based materials such as biochar and activated carbon; mineral-based carriers like red mud, kaolin and montmorillonite; and organic framework support material (MOF) carriers. Among these, biochar carriers have become a research focus due to their low cost and unique advantages in solid-waste resource utilization [30,31,32].

Biochar (BC) inherently has a high specific surface area and abundant surface functional groups [33]. As an ideal supporting material, it can significantly improve the dispersibility of nZVI and effectively inhibit nanoparticle aggregation and oxidative deactivation [34]. At the same time, biochar acts as an electron mediator that accelerates electron transfer between nZVI and pollutants and promotes reductive dechlorination efficiency. The porous structure of biochar alleviates nZVI aggregation and passivation. High-loading biochar-supported nZVI and achieved 87.77–98.50% removal for methylene blue, rhodamine B and tetracycline in single-pollutant systems [29]. Iron-modified base-activated biochar exhibited a removal efficiency of 92% for imidacloprid (IMI) when the initial IMI concentration was 23.8 mg/L and adsorbent dose was 2.5 g/L [35].

After loading with nZVI, organic pollutants adsorbed and enriched inside pores can be reduced by electrons donated from Fe^0^. In this process, Fe^0^ is oxidized, and target pollutants accept electrons to achieve reduction [36]. This can achieve an adsorption–degradation–regeneration cycle. nZVI-BC can remove organic pollutants through synergistic adsorption and catalytic degradation [37]. Biochar plays dual role in the dechlorination of TCE catalyzed by nZVI/Ni bimetallic catalysts. On one hand, its large specific surface area and abundant surface functional groups promote the dispersion of Fe/Ni nanoparticles and inhibit aggregation. On the other hand, its high electrical conductivity enhances the electron transfer capability of the bimetallic catalyst and promote the generation of reactive atomic hydrogen. Highly reactive catalysts BCs@FN were developed using BC loaded with nZVI/Ni (FN) nanoparticles. When the dosage of BC900@FN is 1 g/L, BC900@FN can achieve complete dechlorination of 20 mg/L TCE within 3 h, and the main products are ethane and ethylene [6]. Compared to bare nZVI, BC-nZVI exhibits a higher specific surface area and TCE degradation efficiency. More importantly, the electrochemical properties of biochar lower the corrosion potential of nZVI, enhance the electron transfer capability and promote direct electron transfer for TCE degradation. Concurrently, the enhanced electrochemical performance promotes the reaction between nZVI and water and sustains the continuous generation of atomic hydrogen, which plays a key role in reductive dechlorination [38].

nZVI-BC composite is recognized as a novel remediation material that combines high efficiency and environmental friendliness, with broad application prospects in water treatment and soil remediation. However, attention must be paid to control the loading amount. Insufficient loading leads to an inadequate number of active sites and limited degradation efficiency, while excessive loading causes nZVI particles to aggregate within the pores, which can block carrier channels and reduce the adsorptive capacity for pollutants.

### 2.4. Sulfidation Modification

**Figure 3:**
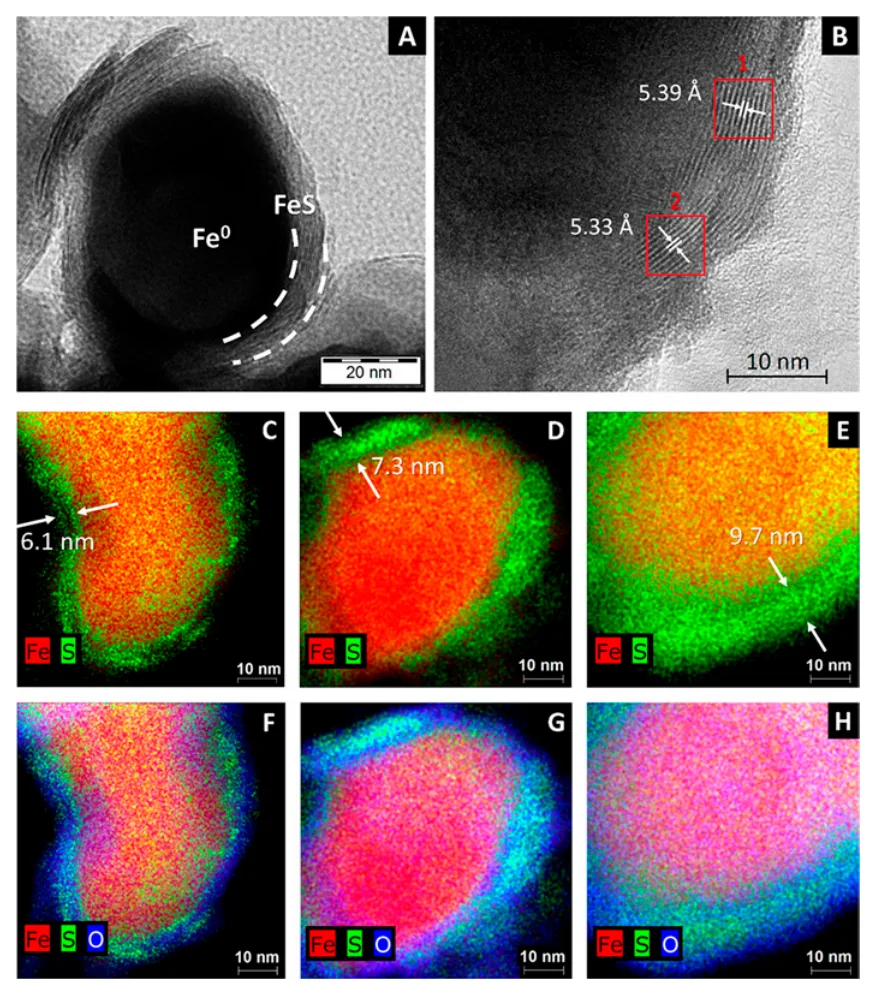
(**A**) TEM image of a s-nZVI particle with a S/Fe mass ratio 0.0195. (**B**) High-resolution transmission electron microscopy detail on the sulfide shell structure of a S-ZVI particle with a S/Fe mass ratio of 0.0094. EDS mapping of S-nZVI particles at S/Fe ratios of 0.0094 (**C**,**F**), 0.0195 (**D**,**G**) and 0.0629 (**E**,**H**). Reproduced with permission from reference [39].

Sulfidation has become a research hotspot for nZVI modification. An FeS layer is formed on nZVI surface via one-step or two-step methods. The synthesized sulfidated nanoscale zero-valent iron (S-nZVI) also exhibits a core–shell structure, wherein the core is dominated by zero-valent iron, while the shell is primarily composed of amorphous iron sulfides (Figure 3) [39]. Such modification markedly improves material electron transfer performance and hydrophobicity.

Extensive studies have been conducted on sulfidation modification and its composite modification strategies. Sulfur incorporation reduces local Fe vacancies and lowers electron transfer resistance. By controlling the sulfur loading and morphology, S-nZVI achieves up to 70% electron efficiency for TCE degradation [40]. S-nZVI achieved a 12-fold higher TCE removal rate and a 7-fold improved electron efficiency compared with pristine nZVI [39]. TCE can be rapidly transformed into ethane and ethylene without intermediate accumulation, providing a safe environment for subsequent microbial degradation [41]. Cyclic voltammetry and EPR revealed that unmodified nZVI tends to convert active hydrogen produced from water reduction into inert adsorbed state or H_2_, whereas S-nZVI retains more surface-adsorbed active hydrogen, thereby suppressing H_2_ evolution and diverting more electrons toward pollutant reduction [42]. S/P-nZVI prepared by one-step liquid-phase reduction undergoes lattice expansion and the multiple Kirkendall effect induced by sulfur doping, generating internal radial nanocracks that act as electron transport pathways. The FeS layer inhibits hydrogen evolution reaction sites and relieves nZVI passivation. S/P-nZVI exhibited a dechlorination rate constant of 0.65 h^−1^ and an electron efficiency of 14.5%. It demonstrates excellent stability in the presence of common groundwater contaminants including PCE, TCE, carbon tetrachloride and carbon trichloride [43]. The FeS shell formed on nZVI’s surface makes the material relatively hydrophobic, with an increased water contact angle. This hydrophobic property can slow the corrosion of the material caused by water and dissolved oxygen, thereby improve reaction persistence. At the same time, the FeS protective shell can block external air and protect the internal Fe^0^ from oxidative passivation [44]. Introducing a second metal on the basis of sulfidation modification can construct metal-loaded, sulfidated nanoscale zero-valent iron (Me/S-nZVI). Compared to single sulfidation modification, Me/S-nZVI shows significantly enhanced TCE degradation efficiency via β-elimination and hydrogenolysis pathways [16]. It is worth noting that sulfur modification can reduce nZVI biotoxicity and make the modified material more environmentally friendly [45].

Sulfidation technology offers certain advantages. FeS_x_ possesses a much narrower bandgap than FeO_x_, enhancing electron selectivity and facilitating electron transfer from Fe^0^ to target organics [40]. The FeS_x_ layer can block the contact between H_2_O and Fe^0^/FeO_x_ sites and reduce electron waste caused by hydrogen evolution reactions [42]. It can also improve the hydrophobicity of the material. Surface FeS_x_ reduces the interaction between the material and water molecules and mitigates material passivation [44].

There are still challenges in current sulfidation techniques. The synthesis stage inevitably generates toxic H_2_S. An inappropriate S/Fe ratio can lead to the formation of an overly thick sulfidation layer, which acts as a physical barrier. Excessive sulfidation can block electron transfer and reduce the reactivity of the material. It not only hinders electron transfer but also restricts the diffusion of reactants, which ultimately impairs the degradation efficiency of the entire system.

### 2.5. Biological Integration Modification

The combination of nZVI and microbial remediation technology is an emerging research direction. Composite systems integrating modified nZVI and microbial communities establish synergistic remediation platforms combining chemical reduction and biomineralization. This system leverages both the strong reductive capacity of nZVI and the low-cost, long-lasting degradation advantages of microorganisms. A synergistic effect can be achieved via both direct or indirect interactions. For direct contact mode, nZVI is mixed with microorganisms, acting as an electron donor to stimulate functional microbial activity. For indirect contact mode, nZVI and microorganisms are deployed separately to achieve functional complementarity through setups such as permeable reactive barriers.

nZVI performs multiple functions within the system. It supplies electrons for microbial extracellular respiration and promotes microbial growth and reproduction. It rapidly reduces highly toxic persistent organic pollutants by cleaving halogen bonds and aromatic rings, transforming these pollutants into low-toxicity, easily biodegradable intermediates for subsequent microbial degradation. It regulates and restores environmental redox potentials to optimize the microenvironment for microbial survival and enhance the stress resistance of microbial communities [46]. Fe^2+^ released from nZVI corrosion can serve as a nutrient source for microbial metabolism [47]. Microbially secreted extracellular polymeric substances alleviate surface passivation of nZVI and accelerate electron transfer [48]. Meanwhile, microorganisms completely mineralize the intermediates generated by nZVI reduction into CO_2_ and H_2_O and avoid toxic intermediates accumulation. Soil amended with nZVI/BC enriches both iron-oxidizing and reductive dehalogenating microbial communities, which further enhances the removal performance of organic pollutants [49].

**Figure 4:**
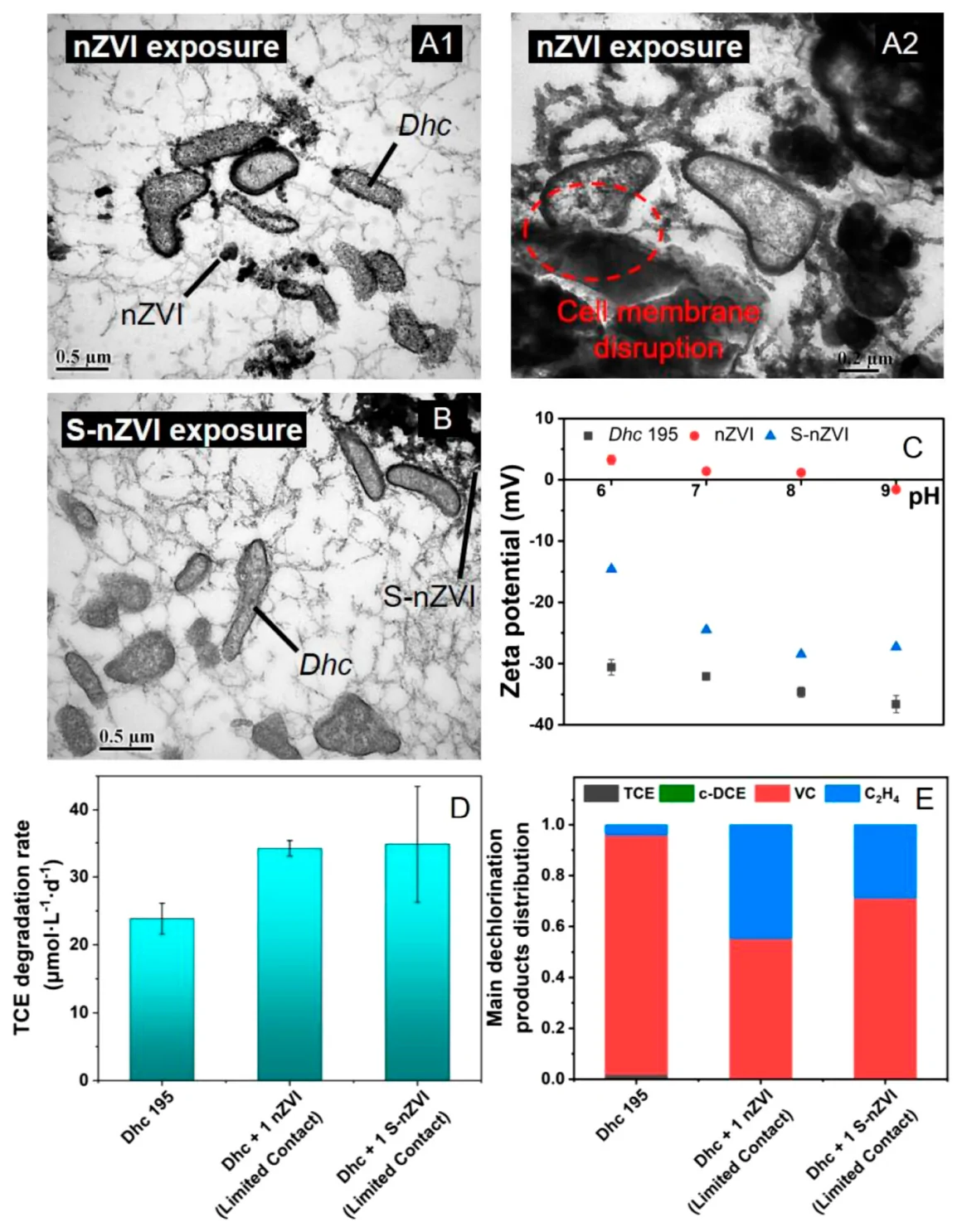
TEM images (**A1**,**A2**) of Dhc 195 with the addition of 1 g/L nZVI; TEM images (**B**) of Dhc 195 with the addition of 1 g/L S-nZVI; zeta potentials of Dhc 195, nZVI and S-nZVI (**C**) in 1 mM NaCl solution across different pH levels; effects of limited contact addition of 1 g/L nZVI and 1 g/L S-nZVI on TCE dechlorination activity (**D**) and dechlorination products (**E**) of Dhc 195 (second TCE degradation cycle). Reproduced with permission from Reference [50], Copyright 2025 American Chemical Society.

Microbial communities are universally present in natural water bodies and soils, and they form a bidirectional regulatory coupling system with modified nZVI. It exhibits both synergistic enhancement and loss-inhibition effects. A high dosage of nZVI exerts cytotoxicity via cell membrane disruption and Fe^2+^-induced oxidative stress, thereby suppress both microbial viability and dechlorination capacity [51]. Surface modification reduces nZVI biotoxicity and enhances its biocompatibility with dechlorination consortia. For example, S-nZVI mitigates the cytotoxicity of *Dehalococcoides mccarty* strain 195 (Dhc 195) by lowering contact toxicity and inherent chemical reactivity, therefore achieving superior dechlorination performance (Figure 4) [50]. The introduction of nZVI/BC into soil promotes the development of microbial communities capable of iron oxidation and reduction-based dehalogenation, thereby significantly enhancing organic pollutant removal efficiency [49]. Combined systems containing *Dehalococcoides* and S-nZVI particles realize sustainable PCE dechlorination under repeated spiking [52]. In nZVI–microbial synergistic systems, the relationship between nZVI dosage and microbial activity is nonlinear. Thus, the nZVI dosage should be optimized to achieve synergistic effects.

Biologically integrated modification offers several advantages. Through the synergistic degradation of chemical and biological processes, pollutants can be completely mineralized without accumulation of toxic intermediates. It requires low material dosage and carries minimal secondary pollution risk. It is suitable for the long-term in situ soil and groundwater remediation with low maintenance costs.

There are also certain limitations. Long reaction cycles and slow microbial metabolism result in insufficient treatment efficiency for high-concentration industrial wastewater. Microorganisms are sensitive to pH, temperature and coexisting toxic substances, which leads to a narrow range of environmental adaptability. The microbial acclimation at the site and the storage process of composite systems are complex.

### 2.6. Synergistic Modification Strategy

Each individual modification approach has inherent limitations. Combining two or more modification strategies enables complementary advantages and synergistic effects [32,53]. Composite modification simultaneously achieves multiple functions including particle dispersion, optimized electronic selectivity, long-term anti-passivation performance, targeted pollutant accumulation and bio-cooperative mineralization. Targeted design of multi-strategy composites will become a core route for developing high-performance nZVI remediation materials.

Representative composite-modified nZVI materials are summarized as follows: Sulfur- and phosphorus-co-modified nZVI (S/P-nZVI) delivers a TCE dechlorination rate constant 20.9 times higher than pristine nZVI [43]. CS-supported and CMC-coated CS@nZVI-CMC ternary composites can simultaneously removal multiple pollution. A removal efficiency of 98.52% for the organochlorine pesticide dichlorodiphenyldichloroethylene (DDE) and 94.01% for heptachlor was achieved via adsorption and electron transfer mechanisms [54]. Carboxymethyl chitosan (CMCS) and CMC can act as stabilizers for S-nZVI. Surface functional groups form covalent bonds with S-nZVI and improve particle dispersibility for contaminated nitrobenzene degradation [55]. Montmorillonite-loaded S-nZVI (MMT/S-nZVI) exhibited maximum adsorption capacities of 22.3 mg/g for chlorinated polyfluoroalkyl ether sulfonate (F-53B) [32]. Bentonite-supported nZVI (Bent-nZVI), further modified with cetyltrimethylammonium bromide (CTMAB) to form Bent-nZVI/CTMAB, raises dichlorophenol removal efficiency from 7.6% to 66.6% [56]. A S-nZVI-PUF composite carrier was developed by loading S-nZVI into polyurethane foam (PUF). This composite prevents the rapid aggregation and passivation of SnZVI while enhancing its efficiency in removing organic compounds [57]. S-nZVI loaded on straw shell biochar (S-nZVI@SS) exhibits remarkable reductive degradation performance toward tris(2-chloroethyl) phosphate (TCEP) in groundwater environments [53]. Biochar-supported S-nZVI (BC-S-nZVI) composites reduce aquatic organism toxicity and mitigate Acid red 73 (AR73) inhibition toward soil microorganisms [58]. BC@nZVI/Ni completely converts TCE into ethane and ethylene [6]. Cellulose nanocrystal (CNC)-modified CNC-S/nZVI(in) achieved 96.5% tetrabromobisphenol A (TBBPA) removal efficiency at an initial concentration of 10 mg/L with 0.5 g/L material dosage [11]. All the above research validates synergistic enhancement by combined modification. Multi-functional composite coatings integrate merits of different modification routes and exhibit promising application prospects for simultaneous high-efficiency multi-pollutant removal in water treatment.

In summary, different modification approaches exhibit varying effectiveness in removing pollutants. The removal efficiencies of representative modified nZVI for organic pollutants are summarized (Table 1). nZVI performance is jointly governed by multiple structural parameters. These include Fe^0^ core availability, oxide/sulfide shell thickness, FeS_x_chemical composition, S/Fe molar ratio, particle aggregation degree, surface hydrophobicity, surface charge, support porosity, electron transfer resistance and passivation tendency. A too-thick FeS shell originating from excessive sulfidation will block electron transfer and suppress reactivity. A moderate S/Fe ratio balances hydrophobicity and electron transport. Severe particle aggregation reduces effective active sites. High hydrophobicity alleviates water-induced corrosion, while appropriate surface charge benefits pollutant capture. Porous supports can improve dispersion, but over-loading will trigger pore blockage. Passivation consumes the Fe^0^ core and terminates remediation reactions. Rational regulation of the above structural parameters is critical for preparing nZVI-based materials with high reactivity and excellent pollutant selectivity.

## 3. Action Mechanisms of Modified nZVI in Organic Pollutant Removal

Modified nZVI composites possess efficient adsorption, reduction and catalytic properties, which enable the effective removal of various organic pollutants. Organic pollutants removal by modified nZVI was the synergistic effect of multiple physical and chemical reaction. And the dominant reaction pathways vary across different modification systems. Removal mechanisms of modified nZVI toward organic pollutants mainly depend on the nZVI surface properties and organic pollutant type. Sulfidation and bimetallic modification are centered on direct reduction dehalogenation. The activated persulfate and molecular oxygen systems depend on advanced oxidation radical mineralization. A carrier-loaded modification system relies primarily on carrier adsorption coupled with interfacial redox reactions. A bio-coupled modification system combines reduction, oxidation and microbial mineralization. This review primarily examines three key mechanisms: direct reduction degradation, advanced oxidation degradation and adsorption–degradation synergy.

Multiple experimental tools are applied to decipher reaction mechanisms. Radical quenching experiments combined with electron paramagnetic resonance are used to identify reactive oxygen radicals, after which the detection of degradation intermediates and elemental mass balance is conducted. Total organic carbon measurement is adopted to determine the mineralization degree and monitor the consumption of Fe^0^. Surface spectroscopy techniques including XPS, Mössbauer spectroscopy, XANES and EXAFS are used to analyze surface speciation transformation.

### 3.1. Direct Reductive Degradation Mechanism

For readily reducible pollutants, direct reduction by nZVI is the primary degradation pathway. As a strong reducing agent, Fe^0^ directly transfers electrons to pollutant molecules, resulting in contaminants’ reductive removal.

**Figure 5:**
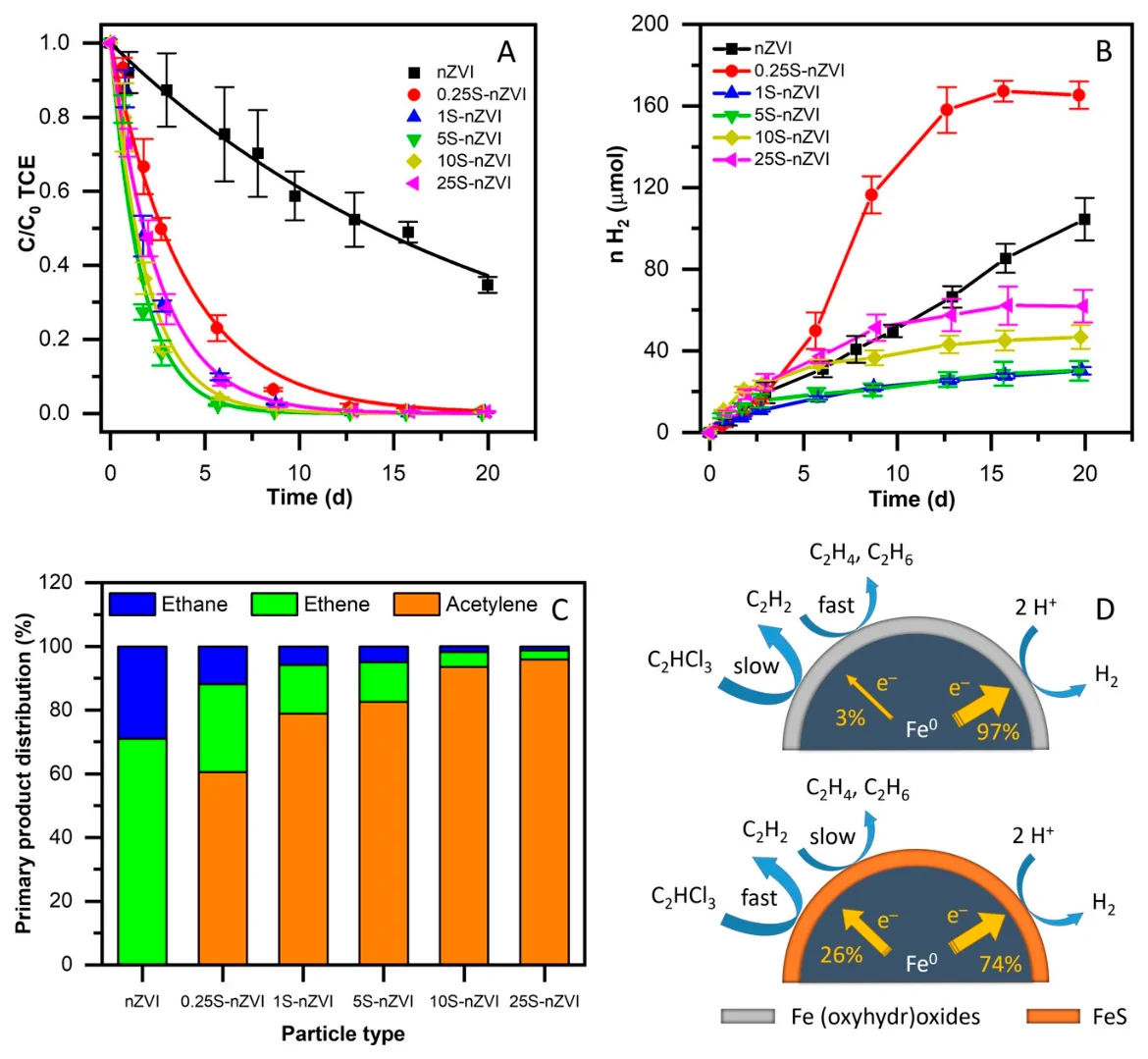
TCE dechlorination by pristine nZVI and S-nZVI with varying S/Fe ratios: (**A**) TCE removal, (**B**) hydrogen production, (**C**) distribution of primary products and (**D**) schematic of the proposed mechanism of TCE dechlorination and hydrogen evolution. Reproduced with permission from reference [39].

Halogenated hydrocarbons are the most common persistent organic pollutants in water bodies. The removal of these pollutants by modified nZVI primarily relies on the reductive dehalogenation mechanism. Some modification strategies can achieve a synergy between reductive dechlorination and catalytic degradation [64]. During the reductive dechlorination process, nZVI acts as an electron donor and transfers electrons toward halogen atoms to cleave C-X bonds. Halogen atoms are substituted by hydrogen atoms, and pollutants are eventually transformed into non-toxic or low-toxic hydrocarbons (Figure 5C,D) [39]. Pristine nZVI exhibits a relatively slow reductive dechlorination rate, whereas modified nZVI can significantly enhance dechlorination efficiency through metal catalysis and enhanced electron conduction. For example, nZVI/Ni nanocomposites presented higher catalytic activity on TCE than Ni nanoparticles and nZVI due to the co-catalytic effect of Ni [6]. In Pd/nZVI, Pd acts as a catalyst to lower dechlorination activation energy, which accelerates electron transfer from Fe^0^ to halogenated hydrocarbon molecules and promotes their degradation [65]. Pd/nZVI can achieve rapid and complete dechlorination of TCE and polychlorinated biphenyls into non-toxic ethylene and ethane, achieving complete detoxification of the pollutants [8]. Compared with pristine nZVI, S-nZVI particles exhibit higher electronic efficiency during the TCE dechlorination reaction. S-nZVI samples with different S/Fe ratios all achieved complete TCE degradation and chlorination conversion (Figure 5A,B) [39]. Product distributions reflect the dechlorination pathway by modified nZVI. The products of TCE dechlorination by BCs@FN were identified as Cl^−^, C_2_H_4_ and C_2_H_6_. Among them, C_2_H_6_ was identified as the main product, while the yield of ethylene was lower, indicating that C_2_H_4_ hydrogenation was the dominant reaction. The remarkable linear relationship between Cl^−^ concentration and the molar amount of gaseous products demonstrates that dechlorination and hydrogenation reactions occur simultaneously throughout the entire process [6]. GC-MS analysis identifies that the degradation products of TBBPA include tribromobisphenol A, dibromobisphenol A, bromobisphenol A and bisphenol A. This indicated that TBBPA undergoes a step-by-step debromination process over CNC-S/nZVI(in). The Br^−^ ions were released corresponding to the cleavage of the C–Br bond and the replacement of the Br atom by hydrogen [11].

Nitroaromatic compounds are highly toxic and carcinogenic and are widely distributed in industrial wastewater and soil. Removal of these compounds by modified nZVI primarily follows a reduction mechanism. A highly toxic nitro group is reduced to the less toxic amino group to achieve detoxification and pollutant degradation. Its reduction performance is closely related with material electron transfer capacity. Metal modification enhances electron transfer efficiency and accelerates reduction. In Ni/nZVI systems, Fe is oxidized to Fe^2+^ at the anode, while Ni accepts and transfers electrons toward the nitroaromatic compound. Additionally, Ni catalyzes the dissociation of H_2_ generated from nZVI oxidation into H*, which participates in nitro group reduction and improves the reduction efficiency [18].

### 3.2. Advanced Oxidation Degradation Mechanism

nZVI can activate molecular oxygen, hydrogen peroxide or persulfate to generate highly reactive species such as hydroxyl radicals (•OH), sulfate radicals (•SO_4_^2−^), superoxide radicals •O_2_^−^) and singlet oxygen (^1^O_2_) [5,44]. These radicals non-selectively attack organic pollutant molecules and mineralize them into CO_2_ and H_2_O [12].

This process is applicable to a variety of non-halogenated organic pollutants, including dyes, antibiotics and phenols. Existing studies have clarified that the iron oxide shell mediates the molecular oxygen activation mechanism. The iron oxide interface catalyzes the single-electron reduction of O_2_ to superoxide radicals and triggers a chain reaction that continuously generates reactive oxygen species [2]. CDP-nZVI efficiently activates persulfates and continuously treats naphthalene-contaminated river water via continuous flow processes. Transformation-product identification coupled with ECOSAR-based toxicity assessment demonstrated that CDP-nZVI/PS degrades naphthalene into low-toxicity phthalic intermediates, which are ultimately oxidized to small-molecule organic acids (tartaric acid and dienoic acid) with negligible secondary pollution risks [23]. Free radical quenching experiments combined with EPR spectroscopy clarify the TC degradation mechanism over NZVI@C-ZSM. Results indicates that hydroxyl radicals (•OH) and superoxide radicals (•O_2_^−^) participate in the reaction process, and the effect of •O_2_^−^ is greater than that of •OH [26].

Research of biochar-supported nanoscale zero-valent iron oxidatively degrading rhodamine B and tetracycline shows that radical oxidation completely disrupts both the chromophoric groups of the dye and the ring structures of the antibiotic, which enables complete decolorization and detoxification [29]. Natural organic matter such as humic acid can inhibit Fe(II) precipitation by forming Fe(II)-HA complexes, thereby increasing the yield of •OH by several times [27]. Theses finding are of great significance for understanding the oxidative degradation behavior of nZVI in natural water bodies. Degradation products are small-molecule organic acids or complete mineralization to CO_2_ and H_2_O. Compared with direct reduction, the advantage of the advanced oxidation pathway lies in its ability to completely mineralize pollutants without generating toxic intermediates [5,20].

### 3.3. Adsorption–Degradation Synergistic Mechanism

For certain organic pollutants that are difficult to directly reduce or oxidize, an adsorption–degradation synergistic mechanism plays a dominant role. Biochar, cyclodextrins and MOF porous carriers pre-enrich organic pollutants through multiple molecular interactions. They exhibit multiple adsorption mechanisms toward organic pollutants, including π–π conjugation, electrostatic interactions and hydrogen bonding [66,67]. Aromatic organic pollutants and polycyclic aromatic hydrocarbons are adsorbed through π–π conjugation. Charged antibiotic molecules are bound by electrostatic interactions. Polar organic pollutants form complexes via hydrogen bonding. Small-molecule organic compounds are captured via microporous filling. Cyclodextrins specifically sequester hydrophobic persistent organic compounds through their hydrophobic cavities [23,28]. TBBPA adsorption follows the chemisorption process via electron transfer or electron pair sharing. It could be adsorbed to the CNC-S/nZVI(in) surface through electrostatic interaction, van der Waals force and the occurrence of surface hydrogen bonding [11]. Carriers concentrate pollutants in local regions of the active interface, which significantly increases the pollutant concentration at the interface and accelerates the kinetics of reduction and oxidation reactions. Simultaneously, the carrier adsorbs humic acids and inorganic ions from the water and mitigates the passivation and inhibitory effects of coexisting components on nZVI.

nZVI-BC composite is a typical representative of the adsorption–degradation synergistic mechanism. The high specific surface area and abundant pore structure of biochar can efficiently adsorb organic pollutants [49]. The adsorbed pollutants are in close contact with the supported nZVI, which facilitates electron transfer and radical attack. Additionally, the graphitized structure of biochar can act as an electron mediator, which promotes electron transfer between Fe^0^ and pollutants [37,68]. In ZVI-BC composites, BC primarily serves to enhance pollutant adsorption. ZVI plays a dominant role in degradation. It mainly degrades organic compounds through reduction and oxidation mechanisms [69,70]. Pure biochar only achieves pollutant immobilization via adsorption. After biochar is loaded with nZVI, it enables in situ degradation of saturated pollutants and regeneration of carrier pores, which overcomes the issue of adsorption material saturation failure [33]. nZVI-loaded biochar efficiently treats complex dyeing wastewater. Its pores adsorb methyl blue, rhodamine B and tetracycline, while pore-confined nZVI drives reductive oxidative degradation [29]. BC-nZVI exhibits higher TCE degradation efficiency. The electrons generated by nZVI can either directly attack TCE or be transferred to TCE through the biochar carrier. Hydrogen radical generation plays a pivotal role in the reductive dechlorination process of BC-nZVI. Compared to lignin-based biochar-supported nZVI (LBC-nZVI), coconut shell-based supercapacitor biochar-supported nZVI (CSBC-nZVI) and coconut shell-based biochar-supported nZVI (CBC-nZVI) demonstrate significantly enhanced H* production capacity [38]. BC-nZVI eliminates halogenated hydrocarbons via synergistic adsorption–dechlorination. BC physically adsorbs and enriches target contaminants at nZVI surfaces to enhance contact with active sites. nZVI further delivers electrons to trigger reductive dechlorination [38]. XPS confirms that nZVI is partially oxidized into iron hydroxides, as evidenced by the increased content of Fe-OH. The weakened Fe^0^ peaks and strengthened Fe^2+^/Fe^3+^ signals after the reaction verify that Fe^0^ is oxidized during reductive dechlorination. In this process, BC accelerates electron transfer and stabilizes the active iron sites, thus maintaining the adsorption–reduction synergy for PCE elimination [30]. Transition metal-modified nZVI can accelerate electron transfer through the galvanic cell effect while catalyzing the decomposition of H_2_O_2_ to generate •OH, which achieves a synergy between reductive dechlorination and oxidative degradation of halogenated hydrocarbons [20]. However, it is noteworthy that the presence of degradation products may influence the degradation mechanism.

## 4. Key Factors Affecting the Reaction Activity of Modified nZVI for Pollutant Degradation

The reaction activity of modified nZVI for organic pollutant degradation is primarily governed by three categories of factors: first, intrinsic material properties related to the interface structure, including oxide layer thickness, modification type, loading amount and sulfide layer composition; second, external environmental factors involving coexisting components such as inorganic ions, natural organic matter and dissolved oxygen; and third, external operational parameters including system pH, reaction temperature, initial pollutant concentration, material dosage, etc.

### 4.1. Intrinsic Properties of the Material

Factors such as the particle size, specific surface area, surface functional groups, thickness and chemical composition of the oxide layer and sulfidation degree of modified nZVI directly affect its reaction activity toward organic pollutant degradation [7]. Generally, a smaller particle size and larger specific surface area provide more reactive sites and lead to higher degradation efficiency. However, excessively small particle size can exacerbate aggregation, thus reducing the effective reaction area.

The thickness and chemical composition of the oxide shell layer in modified materials are pivotal factors that influence degradation performance. An excessively thick shell layer completely blocks electron transfer and leads to material passivation. Conversely, an overly thin shell layer predisposes particles to agglomeration and rapid oxidation by dissolved oxygen in water. The iron oxide shell layer is the critical structural component that determines the reactivity of nZVI. It has an optimal thickness threshold. Shells thinner than 3 nm cause particle agglomeration and rapid deactivation by dissolved oxygen. Shells of 3–8 nm enable electronic conduction, stabilize particle structure and activate molecular oxygen for radical generation and deliver optimal degradation efficiency [2]. Variations in shell layer chemistry also significantly impact performance. Fe_3_O_4_-dominated layers exhibit semiconducting conductivity and low electron transport resistance. The FeOOH hydroxide shell provides a dense insulating layer that rapidly passivates the particles. This is the core mechanism by which sulfide modification improves long-term performance by replacing the hydroxide shell with an FeS layer.

**Figure 6:**
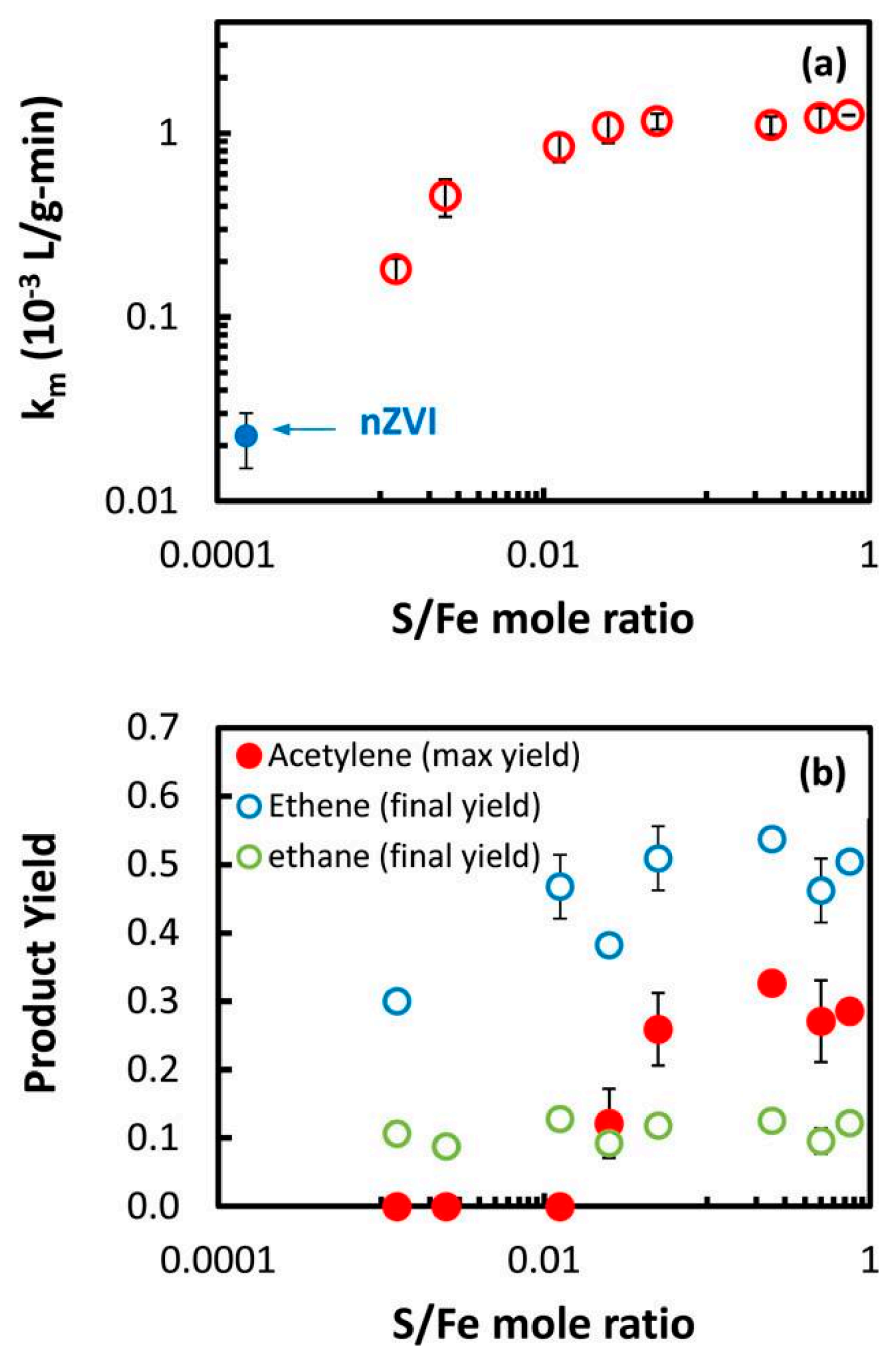
Effect of S/Fe mole ratio on (**a**) TCE degradation rate and (**b**) product yields. Reproduced with permission from reference [64], Copyright 2016 American Chemical Society.

Different modification technologies and process parameters directly regulate oxide shell layer formation and interface properties. Metal modification creates electronic conduction pathways in the oxide layer, which mitigates the inhibitory effect of thick oxide layers. However, insufficient noble metal deposition results in a weak galvanic cell effect, while excessive deposition covers Fe^0^ active sites and reduces reduction activity. Surface coating modification isolates oxygen from water. The dosage of the coating agent determines the porosity of the coating layer, while excessive coating blocks electron transfer [32]. Carrier loading modification restrains thick FeOOH passivation layer formation, where the nZVI loading amount and carrier pore structure are the key parameters. Excessive loading causes pore blockage and particle aggregation, while insufficient loading leads to an inadequate number of active sites [32]. Sulfidation modification directly replaces the oxide shell with a conductive FeS interface and optimizes shell electronic conduction efficiency. The S/Fe molar ratio determines FeS layer thickness. Insufficient sulfidation only leads to limited improvement in electron selectivity, whereas excessive sulfidation impedes electron transport [39,71]. The reactivity of S-ZVI toward pollutants strongly depends on the S/Fe ratio. S-nZVI with a moderate sulfidation degree typically exhibits the best electron selectivity and reactivity [40,44]. At different S/Fe ratios, the Lindane removal efficiency of S-nZVI@BC first increased and then decreased. When the S/Fe ratio was 0, 0.032, 0.060, 0.077, 0.117 and 0.130, the corresponding Lindane removal efficiencies were 41.87%, 71.18%, 96.07%, 99.99%, 78.88% and 65.44%, respectively [31]. The S/Fe ratio significantly influences both the degradation rate of TCE and product accumulation [64]. When the S/Fe ratio is less than 0.025, the dechlorination rate of TCE increases as the S/Fe ratio rises. However, once the S/Fe ratio exceeds 0.025, the reaction rate constant approaches a limiting value (Figure 6a). For particles prepared under low-sulfur conditions, no acetylene was detected in the product mixture, whereas particles with higher sulfur concentrations exhibited significant acetylene accumulation before its conversion to downstream products (Figure 6b). Sulfide modification not only significantly enhances the performance of nZVI in degrading organic compounds but also minimizes the formation of toxic by-products [72].

### 4.2. Environmental Conditions

Environmental factors including dissolved oxygen, coexisting anions and organic matter exert influences on modified nZVI reaction activity [27].

**Figure 7:**
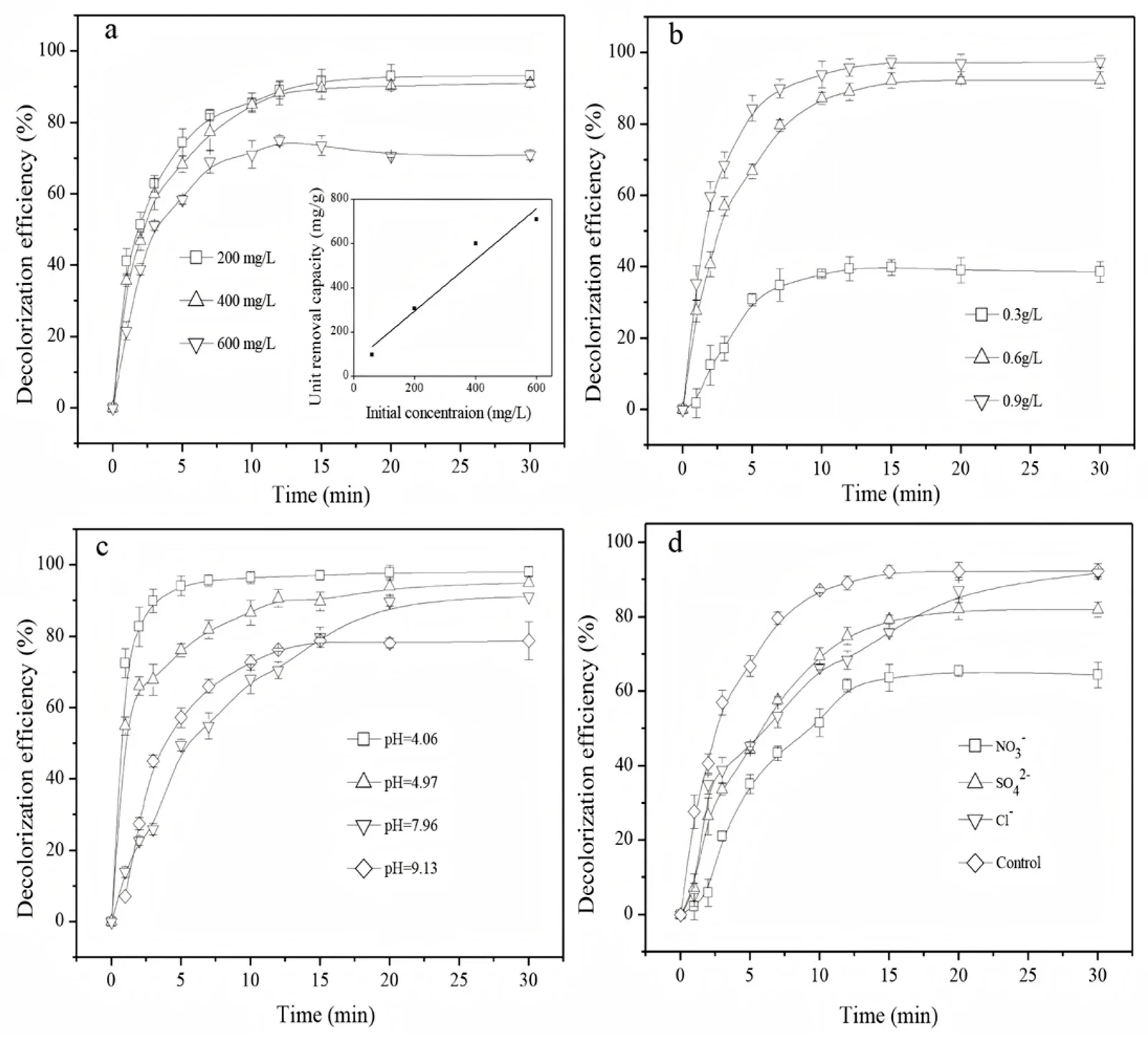
Factors affecting removal of MO: (**a**) different initial concentrations; (**b**) various nZVI/BC_5_ dosages; (**c**) various pHs; and (**d**) various anions. Reproduced with permission from reference [69].

Ionic components in water exert a dual regulatory effect on the activity of nZVI, and final outcomes depend on ion concentration and modification types [4]. Cations such as Ca^2+^, Mg^2+^ and Na^+^ compress the electric double layer on the surface of nZVI particles, weaken electrostatic repulsion, exacerbate particle aggregation, reduce exposed active sites number and lower degradation efficiency. High-valent cations exhibit stronger inhibitory effects than low-valent cations. Cyclodextrin coating or biochar loading modification can mitigate cationic aggregation through spatial hindrance [23]. Different anions exhibit distinct influence mechanisms. PO_4_^3−^ and CO_3_^2−^ react with Fe^2+^ and Fe^3+^ to form insoluble iron salt passivation layers covering particle surfaces. NO_3_^−^ is reduced by nZVI and consumes reactants. Low concentrations of SO_4_^2−^ can be activated by nZVI to generate sulfate radicals that synergistically oxidize organic pollutants. The FeS layer on S-nZVI resists anionic precipitation passivation, which demonstrates significantly superior interference resistance compared to native nZVI. S-nZVI/BC activates persulfate to degrade CIP. The impact of coexisting anions on CIP degradation efficiency is ranked as CO_3_^2−^ > NO_3_^−^ > SO_4_^2−^ > Cl^−^ [73]. Cl^−^ had almost no effect on the final methyl orange (MO) removal efficiency achieved by nZVI/BC5, whereas SO_4_^2−^ and NO_3_^−^ significantly reduced the decolorization efficiency (Figure 7d) [69].

Dissolved oxygen (DO) exhibits bidirectional effects. On one hand, DO is activated by the nZVI interface to generate highly oxidative free radicals for organic pollutant mineralization. On the other hand, DO continuously oxidizes and consumes the Fe^0^ core and accelerates material passivation and deactivation. These two reactions compete for electrons simultaneously. Sulfidation modification and bimetallic modification improve electronic selectivity and reduce the consumption of ineffective oxidation of Fe^0^. Acetaminophen degradation by sulfated ferrous iron-activated peroxodisulfate demonstrated that sulfated ferrous iron achieves higher acetaminophen removal efficiency under both aerobic and anaerobic conditions. This performance is attributed to the multiple effects of O_2_. It not only accelerates the conversion of Fe^0^ to Fe^2+^ but also significantly competes with PDS for the generated Fe^2+^ [74].

### 4.3. Reaction Environment Operation Parameters

Reaction parameters such as pH, pollutant concentration and material dosage significantly influence the reaction activity of modified nZVI.

pH is critical for nZVI corrosion, the formation of passivation layers and the existence forms of free radicals. An acidic environment accelerates Fe^0^ corrosion, leading to substantial electron release and rapid reduction degradation rates. However, in a strong acidic environment, a large amount of iron ions are released, which accelerates leaching of material and reduces the removal efficiency of pollutants [4]. Under a neutral environment, pristine nZVI rapidly forms an Fe(OH)_3_ passivation layer and loss activity. Sulfidation and carrier loading modification stabilize material performance under neutral conditions and are suitable for groundwater remediation. Alkaline environments, the extensive formation of hydroxide ions, leads to the deposition of a dense iron hydroxide passivation film. This film completely blocks electron transfer and significantly reduces the degradation activity of nZVI systems. Under neutral or alkaline pH conditions, the dechlorination rate constant of pentachlorophenol by S-nZVI increases with rising initial pH due to the formation of a hydroxide layer that enhances adsorption. Conversely, under acidic initial pH, the dechlorination rate constant rises with decreasing initial pH because hydrogen ions diffusing in the solution participate in the reaction [75]. At pHs 4 and 5, BC-S-nZVI achieves high removal efficiency for AR73 within a short time. However, the removal efficiency significantly decreases at pH 8 [58]. At pH 5, S-nZVI exhibits optimal removal efficiency for Lindane. Finally, as the pH increases, the degradation efficiency decreases [31]. As the initial pH increases, the MO removal efficiency exhibits a declining trend. At pH 4.06, approximately 98.51% of dye can be removed. When the pH increased to 7.96, the decolorization efficiency of MO was approximately 91.2% (Figure 7c) [69].

Modified nZVI dosage has an optimal value. Insufficient dosage limits the number of active sites, while excessive dosage may cause particle aggregation and increase treatment costs [8]. S-nZVI@BC (S/Fe]A = 0.077) reaches optimal Lindane removal at a dosage of 2.0 g/L [31]. For nZVI/BC5, only 38.6% of MO exhibited decolorization within the first 15 min at a 0.3 g/L dosage. When the nZVI/BC dosage increased from 0.6 g/L to 0.9 g/L, MO decolorization efficiency increased from 92.1% to 97.3% (Figure 7b) [69]. The reaction rate constant of TCE removal was elevated from 0.46 to 1.97 h^−1^ with the increased dosage of BC900@FN from 0.5 to 2.0 g L^−1^ [6]. TC removal efficiency by nZVI@C-ZSM declines with an increasing initial TC concentration, whereas the actual removed TC amount increased. That is, higher TC initial concentrations inhibit the activity of nZVI@C-ZSM yet enhance its removal capacity [26]. Increased pollutant amounts occupy more reactive sites, which consequently reduces the overall removal efficiency.

The pollutant concentration imposes great influences on the degradation efficiency at a given adsorbent ratio. Generally, the degradation efficiency maintains a high level under low pollutant concentration conditions yet drops sharply at excessively high pollutant loading. For S-nZVI@BC, Lindane is nearly completely removed at initial concentrations of 1 mg/L and 5 mg/L within 24 h, but efficiency decreases drastically when the initial concentration reaches 10 mg/L [31]. MO removal efficiency by nZVI/BC5 decreases with increasing initial pollutant concentrations (Figure 7a) [69]. Under fixed conditions, CMC-PVP-nZVI/Pd achieved nearly complete cyanide removal when the initial cyanide concentration was below 40 mg L^−1^. Removal efficiency gradually decreased from 100% to 58.6% as the cyanide concentration rose to 200 mg L^−1^, which was due to the finite capacity of active binding sites on the nanocomposite [24]. In nZVI–microbe coupled systems, excessively high nZVI doses bring cytotoxicity and weaken microbial activity [76]. For composite organic polluted systems, competition among multiple organic contaminants for adsorption sites causes varied losses in degradation performance. Cyclodextrin-coated nZVI mitigates site competition effects by targeted pollutant capture relying on tunable cavity sizes [23].

Organic pollutant degradation by modified nZVI is a complex interface reaction process involving multiple interacting factors. Intrinsic material activity is determined by particle size, specific surface area, surface functional groups and sulfuration degree. Water chemistry conditions such as pH, dissolved oxygen and coexisting ions regulate interface passivation and electron transfer efficiency. Effective in situ remediation requires comprehensive consideration of these key factors. This includes targeted regulation of the material’s microstructure, optimization of water environment parameters and the selection of appropriate microbial communities to maximize the remediation efficiency of modified nZVI.

## 5. Existing Problems in the Application of Modified Nanoscale Zero-Valent Iron

Although modified nZVI exhibits excellent performance in the field of organic pollutant removal, modified nZVI still faces multiple bottlenecks restricting engineering promotion, reflecting prominent lab-to-field gaps. Most reported conclusions are obtained under static batch laboratory settings. Actual groundwater remediation occurs under dynamic flow-through conditions. Continuous contaminant inflow will change actual treatment performance compared with static tests. Once nZVI particles are injected into aquifer subsurface, physical recovery of nanoparticles is extremely difficult. So, immobilization strategy is essential for field application.

(1) High modification cost: Noble metal modifiers are expensive, which increases preparation costs. Certain modification processes are complex and require specialized equipment, such as a high-energy ball-milling apparatus, hydrothermal reactors, inert-atmosphere reaction setup, vacuum-assisted synthesis, etc. This makes large-scale industrial production difficult.
(2) Insufficient long-term reactivity: Modified material undergoes passivation and activity decay after repeated operation cycles. Surface coatings may detach under complex conditions such as high pH and high ionic strength, triggering nZVI re-aggregation and oxidative passivation and reducing reaction activity.
(3) Limited subsurface mobility. Modified nZVI still has a relatively high density. Certain modifiers increase its particle size. Both factors hinder the long-distance particles mobility in groundwater or soil media. Modified nZVI mostly functions around injection points and yields limited remediation radius [8]. Soil-grain adsorption further impedes particle migration and generates heterogeneous remediation. Nanoparticles cannot be easily retrieved after subsurface injection, so immobilization strategies are essential for field deployment.
(4) Secondary pollution risk: Certain modifiers exhibit inherent toxicity and may leach into the environment during remediation, which causes secondary pollution. Additionally, during oxidation processes, modified nZVI generates iron oxides/hydroxides, which may alter the physicochemical properties of water or soil, such as pH and redox potential, and ultimately affect the ecological environment. Ecotoxicity of modified nZVI and corresponding modifiers should be systematically assessed. Regeneration and reuse feasibility also deserve attention.
(5) Ambiguous complex-pollutant remediation mechanisms: For complicated organic-pollutant systems, degradation pathways are intricate. Synergistic effects of different modification strategies and the corresponding electron transfer pathways require further in-depth investigation.
(6) Lab-to-field gap: Most high removal efficiency results are obtained under favorable laboratory conditions with optimized adsorbent pollutant mass ratios. Such experimental data cannot be directly extrapolated to real environment. Natural soil or water conditions, organic matter and competing inorganic ions produce inhibitory effects, and particle aging is inevitable. Although several pilot-scale and field-scale nZVI remediation cases have been documented [77], it is difficult to compare remediation efficiency due to the varying site conditions. Large-scale demonstration projects are still limited.

## 6. Conclusions and Prospects

### 6.1. Main Conclusions

This review summarizes the modification strategies of nZVI and their application mechanisms for organic pollutant removal. The main conclusions are as follows:

(1) Modification strategies including metal modification, surface coating, carrier loading, sulfidation modification and biological integration can effectively overcome the inherent defects of nZVI. Each modification method has its own advantages and disadvantages, while combined modifications can achieve synergistic performance enhancement.
(2) Removal mechanisms of modified nZVI toward organic pollutants include direct reduction, advanced oxidation and adsorption–degradation synergy. Direct reduction is suitable for chlorinated organics and nitroaromatic compounds. Advanced oxidation can achieve complete mineralization of pollutants. And the adsorption–degradation synergistic mechanism is particularly prominent in nZVI–biochar composite systems.
(3) Remediation performance is jointly controlled by intrinsic material properties and external environmental–operational factors. Excessive sulfidation or over-coating will hinder electron transfer and reduce reactivity. Laboratory high-efficiency results strongly depend on adsorbent pollutant mass ratio and experimental conditions, which cannot be simply transferred to field scenarios.

### 6.2. Future Research Directions

Although modified nZVI has achieved significant progress in the field of organic pollutant removal, multiple challenges and opportunities remain for future investigation:

(1) Construct quantitative structure–activity relationships for modified nZVI to achieve rational material design targeting specific pollutants.
(2) Develop high-selectivity-modified nZVI materials for practical mixed contaminated sites, and deeply reveal the competition synergy mechanisms between multiple pollutants.
(3) Further clarify the distinction between material stability and reaction condition stability, and mitigate particle aging and passivation in actual groundwater environments.
(4) Develop low cost, scalable large-batch synthesis routes for modified nZVI to reduce the preparation cost for engineering applications.
(5) Carry out systematic ecological safety evaluations for modified nZVI and its modifiers, and establish safety thresholds for its environmental applications.
(6) Promote more pilot-scale and field demonstration investigations to narrow the lab-to-field gap, and focus on particle mobility, containment and risk control during in situ injection.

## Figures and Tables

**Table 1:** Summary of representative modified nZVI for organic pollutant removal experiment.

Material	Modification Type	Pollutant	Pollutant Concentration	Material Dosage	Reaction Time	Removal Efficiency	Reference
nZVI/Pd	Metal modification	DCE, TCE and PCE	25 mg/L	1.0 g/L	2 h	100%, 100%, 99.9%	[18]
nZVI/Ni	Metal modification	DCE, TCE and PCE	25 mg/L	1.0 g/L	4 h	100%, 74%, 63%	[18]
nZVI/Mn	Metal modification	SMZ sulfamethazine	5 mg/L	75 mg/L	1 h	95%	[59]
CS@nZVI	Surface coating modification	Textile wastewater COD	850.87 mg/L	1.84 g	1 h	85.53%	[25]
nZVI@C-ZSM	Surface coating modification	TC	30 mg/	16.67	1 h	97.75%	[26]
BC-nZVI	Carrier loading modification	TCE	25 mg/L	2 g/L	1 d	>98%	[38]
BC@nZVI	Carrier loading modification	Atrazine	1 mM	0.25 g/L	30 min	93.8%	[60]
BC-nZVI	Carrier loading modification	Pyrene	6 mM	1.2 g/L	1 h	99.4%	[61]
S-nZVI	Sulfidation modification	TCE	20 mg/L	1 g/L	3 weeks	100%	[39]
S-nZVI	Sulfidation modification	TBBPA	20 mg/L	2.3 g/L	2 h	91.4%	[62]
S-nZVI + *Dhc*	Biological integration modification	PCE	110 μM	1 g/L	14 d	100%	[53]
BC900@FN	Synergistic modification	TCE	20 mg/L	1 g/L	3 h	100%	[6]
CNC-S/nZVI(in)	Synergistic modification	TBBPA	10 mg/L	0.5 g/L	2 h	96.5%	[11]
S-nZVI@BC	Synergistic modification	Lindane	5 mg/L	0.5 g/L	24 h	99.95%	[31]
S-ZVI@ BC	Synergistic modification	Tetracycline	20 mg/L	0.15 g/L	30 min	99.05	[63]

## Data Availability

No new data were created or analyzed in this study. Data sharing is not applicable to this article.

## Abbreviations

The following abbreviations are used in this manuscript:

nZVI	Nanoscale zero-valent iron
PVP	Polyvinylpyrrolidone
CMC	Carboxymethyl cellulose
CS	Chitosan
PAA	Polyacrylic acid
TCE	Trichloroethylene
DCE	Tichloroethene
PCE	Perchloroethene
DDE	Dichlorodiphenyldichloroethylene
S-nZVI	Sulfidated nanoscale zero-valent iron
CMCS	Carboxymethyl chitosan
PCS	Polyethylenimine grafted chitosan
TC	Tetracycline
CD	Cyclodextrin
BC	Biochar
FN	nZVI/Ni
IMI	Imidacloprid
CTMAB	Cetyltrimethylammonium bromide
CNC	Cellulose nanocrystals
TBBPA	Tetrabromobisphenol A
PUF	Polyurethane foam
BC-nZVI	Biochar-supported nZVI
LBC-nZVI	Lignin-based biochar-supported nZVI
CSBC-nZVI	Coconut shell-based supercapacitor biochar-supported nZVI
CBC-nZVI	Coconut shell-based biochar-supported nZVI
MO	Methyl orange
